# Controlled mechanochemical coupling of anti-junctions in DNA origami arrays

**DOI:** 10.1038/s41467-024-51721-y

**Published:** 2024-09-10

**Authors:** Fiona Cole, Martina Pfeiffer, Dongfang Wang, Tim Schröder, Yonggang Ke, Philip Tinnefeld

**Affiliations:** 1 Department of Chemistry, Ludwig-Maximilians-Universität München, Butenandtstr. 5-13, München, Germany; 2 Center for NanoScience, Ludwig-Maximilians-Universität München, Schellingstraße 4, München, Germany; 3 Wallace H. Coulter Department of Biomedical Engineering, Emory University, Atlanta, GA USA; 4 Georgia Institute of Technology, Atlanta, GA USA; 5 School of Biomedical Engineering, University of Science and Technology of China, Suzhou, China; 6 Suzhou Institute for Advanced Research, University of Science and Technology of China, Suzhou, China

**Keywords:** Nanobiotechnology, DNA nanostructures, Single-molecule biophysics

## Abstract

Allostery is a hallmark of cellular function and important in every biological system. Still, we are only starting to mimic it in the laboratory. Here, we introduce an approach to study aspects of allostery in artificial systems. We use a DNA origami domino array structure which–upon binding of trigger DNA strands–undergoes a stepwise allosteric conformational change. Using two FRET probes placed at specific positions in the DNA origami, we zoom in into single steps of this reaction cascade. Most of the steps are strongly coupled temporally and occur simultaneously. Introduction of activation energy barriers between different intermediate states alters this coupling and induces a time delay. We then apply these approaches to release a cargo DNA strand at a predefined step in the reaction cascade to demonstrate the applicability of this concept in tunable cascades of mechanochemical coupling with both spatial and temporal control.

## Abstract (web summary)

Allostery is a hallmark of cellular function and important in every biological system, but difficult to artificially mimic. Here, the authors report an approach to study aspects of allostery in artificial systems, using a DNA origami domino array structure which, upon binding of trigger DNA strands, undergoes a stepwise allosteric conformational change that can be monitored by a double FRET probe.

## Introduction

Allostery is defined as the thermodynamic and mechanochemical coupling of binding reactions to remote conformational changes in molecular systems^1–4^. Nature provides us with a large variety of allosteric systems capable of regulating and modulating biological activity. Allosteric networks enable signal transduction^5,6^ and amplification^6–8^, logical gating^9,10^ and cooperative and anti-cooperative behavior^11,12^. Attaining a similar level of control over these processes in laboratory would allow rationally designing and developing biomolecular networks^13,14^. Building artificial systems capable of mimicking allostery therefore represents a major bioengineering goal^15–19^.

**Fig. 1:**
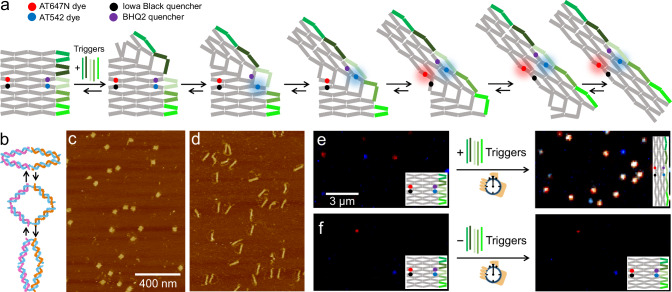
Concept for following the transformation reaction of reconfigurable DNA origami array structures on the single-molecule level in real time. **a** Scheme of the DNA origami array model structure transforming upon addition of DNA trigger strands. Red and green FRET probes (ATTO647N-IowaBlack RQ and ATTO542-BHQ2, red/ black and blue/ purple circles) are placed at the positions at which the transformation reaction is studied. The transformation process occurs diagonally, starting either from the top right corner, as shown in the sketch, or from the bottom right corner. **b** Sketch of conformational flipping of a single anti-junction. Blue DNA strands represent sections of the scaffold strand, whereas pink and orange strands represent different staple strands. **c**, **d** AFM images of the DNA origami array (**c**) before and (**d**) after overnight incubation with trigger strands indicate a successful transformation of the structure. **e**, **f** Exemplary TIRF images of the DNA origami array structure before and after incubation with and without trigger strands. Fluorescence of ATTO647N is shown in red, fluorescence of ATTO542 in blue and co-localized fluorescence of both in white.

Reconfigurable DNA origami array systems have great potential to become a platform to accommodate controlled allosteric cascade reactions over several tens of nanometers (Fig. 1a)^15,17,18^. They consist of multiple equivalent DNA anti-junctions that each exist in two stable conformations between which they can switch through an unstable open conformation (Fig. 1b). Reconfiguration of the whole system is induced by addition of trigger DNA strands that – by hybridization to certain anti-junctions at the edge – stabilize one conformation of the addressed anti-junctions over the other. In a continuous transformation reaction, these anti-junctions relay their conformational information to neighboring anti-junctions causing them to change their conformation. This reaction repeats until all anti-junctions in the array are converted in a diagonal, stepwise, highly coordinated manner. As such, the chemical energy of the trigger DNA strands binding to the structure is first converted to mechanical energy which then propagates through the structure inducing the conformational change.

Our understanding of the cascading transformation in DNA origami arrays has already been greatly improved in previous work where the transformation of DNA structures to different shapes^17,18,20–23^, the initiation of proximity induced operations by the transformation process^24,25^ and the realization of cascaded reactions^26^ were demonstrated. However, the precise nature of the underlying energy landscape and of the transformation kinetics has remained elusive. The lack of knowledge of the mechanism of the transformation at the molecular level prevents us from controlling allosteric behavior in these systems. Therefore, there is a crucial need for new methods to acquire a deeper understanding of the energy landscape and coupling between individual anti-junctions in the transformation process. Can the coupling be altered and how does it influence the timing of the transformation as well as the possibility to create functional devices from reconfigurable DNA origami array systems?

So far, the transformation process was verified by atomic force microscopy (AFM), gel chromatography and gel chromatography combined with ensemble fluorescence measurements^18,27^. Gel chromatography analyzes only the start and the end point of the transformation process^18,20–22,27^, while AFM imaging throughout the transformation process can reveal intermediate states. These states indicate a diagonal transformation pathway that minimizes the number of simultaneously open, unstable conformations of anti-junctions. However, AFM cannot reveal the actual transformation kinetics and pathways, due to the low temporal resolution of AFM and the interference from DNA-mica and DNA-cantilever interactions^18,22^.

Here, we establish a fluorescence-based single-molecule assay to measure the time it takes for the transformation to propagate from one specific anti-junction to another non-invasively. To this end, two pairs of FRET probes were placed on two selected anti-junctions in the reconfigurable DNA origami array system. The FRET probes report on the conformation of the anti-junctions they are placed on which allows measuring their transformation times. Comparing the transformation times at different anti-junctions in single structures revealed the propagation process independent of induction (binding), diffusion or experimental synchronization commonly required by classical chemical kinetics measurements. What’s more, the free selection of FRET-probe anti-junction combination allowed zooming in into every substep of the transformation process and characterizing it at the single-molecule level additionally providing access to subpopulations and kinetic heterogeneity.

Our double-FRET-probe assay provides access to important aspects of allostery in artificial systems and adds timing as an additional dimension. For a small reconfigurable DNA origami model system, we showed that most steps in the transformation cascade are coupled and how their coupling can be influenced by introducing modifications in the structure. Our understanding of the underlying energy landscape was finally used to release a cargo DNA strand at a predefined step in the transformation cascade to demonstrate the applicability of this concept in tunable allosteric reactions.

## Results

### DNA origami array structure as a programmable platform for reaction cascades

We designed a small reconfigurable DNA origami array structure as a model system to establish our assay and to study allostery and mechanochemical coupling in the transformation reaction (Fig. 1a, Supplementary Data 1). The model structure is composed of 5 × 2.5 anti-junctions that can be transformed by hybridization of five trigger DNA strands to the right side of the structure. For the trigger DNA strands, an asymmetric design is chosen that energetically favors the initiation of the transformation reaction at the top right corner rather than at the bottom right corner (see Supplementary Fig. 1). This ensures that the transformation reaction always starts from the same corner, facilitating the direct comparison of transformation times of individual anti-junctions.

AFM imaging confirmed the successful formation of the untransformed DNA origami model structure in a square-like shape when assembled without the addition of trigger DNA strands (Fig. 1c). Overnight incubation with 50 nM of the five trigger DNA strands resulted in the quantitative transformation of the structure into its transformed oblong conformation (Fig. 1d).

As the distances between the individual arms of the anti-junctions change during the transformation reaction, we placed two FRET-pairs as signal transduction elements that report on the transformation at specific locations within the DNA origami. In the FRET-pairs, we used photostable, single-molecule optimized fluorophores as donor dyes in the green and red spectral region (ATTO542 and ATTO647N), respectively. Appropriate dark-quenchers (BHQ2 and IowaBlack RQ) as FRET acceptors were placed such that the donor was strongly quenched before the transformation reaction and lighted up in the moment of transformation. This “turn-on” configuration as exemplarily shown in the sketch of Fig. 1a ensured discrimination of transformation events from photobleaching events. Surface-immobilized structures were imaged via total internal reflection fluorescence (TIRF) microscopy using green and red, alternating excitation (see Supplementary Fig. 1 and Methods for experimental details). In the resulting images, blue, red and white spots represent fluorescence of ATTO542, ATTO647N and co-localized fluorescence of both dyes, respectively.

We recorded TIRF images of DNA origami arrays bearing FRET probes (positions of FRET probes as in Fig.1a) before and after 25 min incubation with and without five trigger DNA strands. The corresponding TIRF images are shown in Figs. 1e, f. Before incubation, the fluorescence of both dyes is quenched. Only a small number of spots is visible, which could be attributed to either mislabeled or partially transformed structures (Figs. 1e, f, left images). After incubation with trigger DNA strands, we noted a significant increase of spots of co-localized fluorescence of ATTO542 and ATTO647N which did not occur after incubation without trigger DNA strands (Figs. 1e, f, right images). This demonstrates that the trigger-induced transformation reaction of the DNA origami array structure occurred and that it could be visualized by fluorescence imaging. With a reference dye for localizing DNA origami structures, we determined the transformation yield as studied with the FRET probes to have values of 86% and 93% depending on the position of the FRET probe (Supplementary Fig. 2).

### Real time single-molecule observation of the transformation reaction pathway of DNA origami arrays

Next, we explored the dynamics of the transformation reaction upon addition of the five trigger DNA strands. To extract dynamic information of the transformation of individual anti-junctions in the structures, we used time-lapse imaging (alternating 638 nm and 532 nm excitation, 100 ms every 1 s per color) on the same area and extracted dual-color fluorescence transients of single structures.

**Fig. 2:**
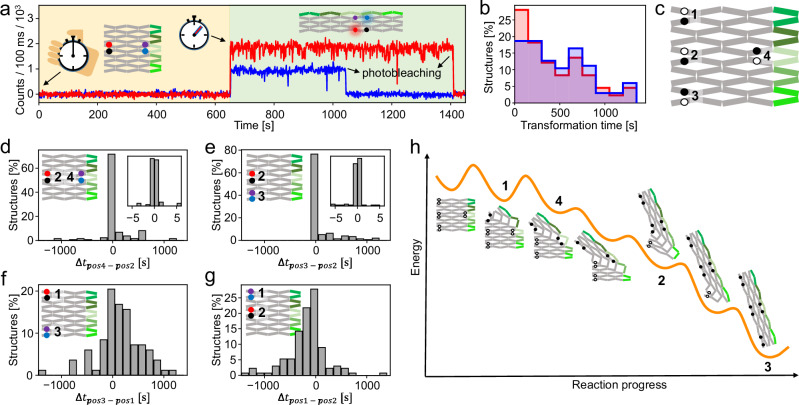
Real-time imaging of the transformation reaction of DNA origami arrays. **a** Representative single-molecule fluorescence intensity transients of DNA origami array with a green and a red FRET probe incorporated after addition of five trigger DNA strands at 0 s. The time the transformation occurs at the red and the green FRET probe positions is marked with an arrow. Fluorescence of ATTO647N and ATTO542 is shown in red and blue, respectively. **b** Transformation time after the addition of five trigger DNA strands at the position of the red FRET probe (red) and the green FRET probe (blue). **c** Scheme of the different positions used for the placement of the FRET probes on DNA origami arrays for tracking the transformation reaction. **d**–**g** Time difference between the transformation occurring at the positions of the green and red FRET probes for different FRET probe positions. **h** Proposed, simplified energy landscape of the transformation reaction. The intermediates at which the studied positions switch their conformation are marked with numbers.

Figure 2a shows an exemplary transient recorded during the transformation reaction (for additional transients, see Supplementary Fig. 3). The transient exhibits a single-step increase in fluorescence intensity occurring simultaneously for both FRET probes within the time resolution of 1 s of our measurement followed by single-step photobleaching. Such an increase was not observable when conducting the same measurement in the absence of the trigger DNA strands, excluding the possibility of it being caused by photobleaching of the quencher molecules (Supplementary Figs. 2j, k). We thus interpreted the single-step increase in fluorescence as the transformation reaction progressing through the corresponding anti-junction and defined the time between the addition of the trigger DNA staples and this increase as the transformation time of the corresponding anti-junction. The transformation times at the positions of the FRET probes were extracted separately for each structure from the transients (Fig. 2b).

To visualize the transformation behavior of different anti-junction combinations, we assembled four different DNA origami array structures bearing red and green FRET probes at different anti-junctions (Positions 1-4, Fig. 2c, Supplementary Fig. 4). We extracted the time differences *Δt* between the transformation occurring at the position of the green and of the red FRET probe for each single construct (Figs. 2d, g). For the FRET probes used for the transient in Fig. 2a, this *Δt* is, for example, 0 s which is the dominating value for the anti-junction combination surveilled by the FRET probes at position 2 and 4 (Fig. 2c, d). Transients and transformation times for the other FRET probe combinations are provided in Supplementary Figs. 3, 5–8).

Time difference distributions of the transformation reactions of DNA origami array structures bearing FRET probes at Position 2 and 4 and at Position 2 and 3 showed a narrow unimodal distribution (Fig. 2d, e). With the exception of a few outlier values, the transformation at the studied positions occurred simultaneously within our temporal resolution of 1 s. In contrast, a time delay between the transformation at Positions 1 and Position 3 was noticed (Fig. 2f). The transformation occurred first at Position 1 and reached Position 3 after an average time of 198 s (see Supplementary Fig. 9 for controls). When the FRET probes were placed at Positions 1 and 2 (Fig. 2g), the transformation again first occurred at Position 1 before progressing to Position 2. In combination, this implies the transformation first occurring at Position 1 before progressing to Positions 2-4 which is in accordance with the intended asymmetric trigger DNA strand design (Supplementary Fig. 1).

Based on our single-molecule fluorescence measurements and previously reported AFM data^18^, we propose a model for the energy landscape of the transformation reaction in our DNA origami array (Fig. 2h). Start and end points of the transformation reaction are the thermodynamically stable transformed and untransformed conformations in which all anti-junctions adopt the same conformation. In all transformation intermediates, some anti-junctions adapt an unstable open conformation. We estimate the energy of those intermediates based on their number of open anti-junctions. The more open anti-junctions a conformation has, the less stable it is. Hybridization of all five trigger DNA strands tilts the energy landscape of the transformation reaction strongly towards the transformed conformation. During the first and second steps of the diagonal transformation reaction, the number of anti-junctions that are forced into their thermodynamically unfavored open conformation increases. Thus, the corresponding steps are accompanied by higher activation energies, resulting in the measured time delay between the transformation occurring at Position 1 and all other positions. In consecutive steps, the number of unstable open anti-junctions remains the same and eventually decreases, which explains the observed quasi-simultaneous transformation at these positions. We then introduced an additional Position 5 which–following our model–transforms in the same step as Position 1. In our measurements, this position transformed at the same time as Position 1 but before Position 3, further confirming the proposed energy landscape (Supplementary Fig. 10).

### Mechanochemical coupled and uncoupled transformation of anti-junctions in DNA origami array structures

**Fig. 3:**
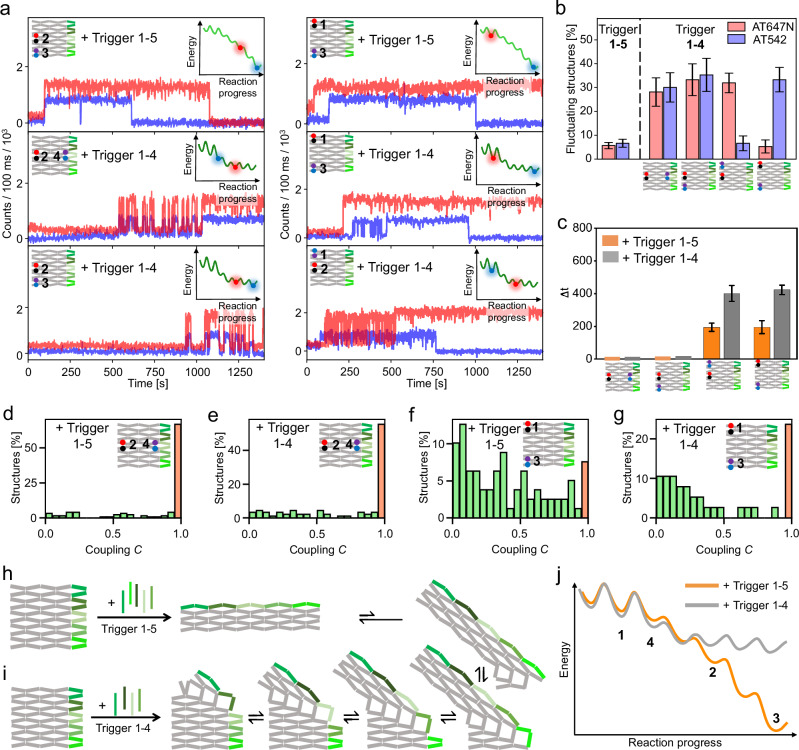
Reversibility and coupling in the transformation reaction upon addition of different numbers of trigger DNA strands. **a** Representative single-molecule fluorescence intensity transients of DNA origami arrays with FRET probes placed at different positions upon the addition of all five trigger DNA strands (upper row) and only the upper four trigger strands (middle and lower row) at 0 s. Fluorescence of ATTO647N and ATTO542 is shown in red and blue, respectively. **b** Fraction of structures exhibiting fluctuations between the untransformed and transformed conformation at the different positions of the FRET probes upon addition of all five or the upper four trigger strands. Error bars represent the standard error of at least 80 structures. **c** Mean absolute time differences for the transformation occurring at the different positions upon addition of five trigger strands and upon the addition of the upper four trigger DNA strands. For designs in which the majority of structures exhibited a time delay between the transformation at the different positions, only non-perfectly coupled structures with $\triangle t\ne 0\,{{{\rm{s}}}}$ were considered. All plots show the mean values and standard errors of Gaussian fits to the corresponding time difference distributions. **d**–**g** Coupling histograms for DNA origami array structures with the FRET probes at different positions. The fraction of structures exhibiting full coupling is indicated by an orange bar. **h**, **i** Scheme of the transformation reaction upon addition of five and the upper four trigger strands. **j** Proposed, simplified energy landscape of the transformation reaction with four and five trigger strands. The potential wells at which the studied positions switch their conformation are marked with numbers.

To study the coupling between the transformation steps and how it can be influenced, we first reduced the driving force of the transformation reaction by reducing the number of added trigger DNA strands from all five to only the upper four. Figure 3a–f shows exemplary single-molecule fluorescence transients of structures with FRET probes placed at different positions. Upon addition of all five trigger DNA strands, over 90% of all transients showed only one irreversible transformation step–independent of the positions of the FRET probes (Figs. 3a, b, and Supplementary Fig. 3, 5–7).

When adding only the upper four trigger DNA strands, a significant fraction of transients showed intensity fluctuations between two well-defined levels for Positions 2–4 (Fig. 3a, Supplementary Fig. 11). In contrast, at Position 1, the fraction of transients exhibiting fluctuations remained at a similarly low level as when adding all five trigger DNA strands (Fig. 3a, Supplementary Fig. 9). We ascribe the emerging fluctuations to reversible transformations of the respective anti-junctions.

A comparison of the transformation times between the studied positions (Fig. 3c, Supplementary Fig. 12) shows the same trends as the transformation upon addition of all five trigger DNA strands. As such, the transformation reaction first irreversibly progresses through Position 1 followed by simultaneous time-delayed reversible transformations at Positions 2-4. However, the time between the transformation occurring at Position 1 and Positions 2-4 increased compared to the time upon addition of all five trigger DNA strands (Fig. 3c). While this indicates that the kinetics of the overall transformation reaction was slowed down by reducing the number of trigger DNA strands added, it did not affect the coupling between anti-junctions to the extent that we could visualize their separate transformations. Further reduction of the number of trigger DNA strands to three or less led to the transformation reaction being either incomplete or not initiated at all (Supplementary Fig. 13).

To further investigate the fluctuating behavior of the anti-junctions upon addition of the upper four trigger DNA strands, we quantified the percentage of fluctuating structures 24 h after addition of the trigger DNA strands. While in many cases, the reversible fluctuations ceased and the fully transformed conformation was adapted in the first 25 min after addition of the trigger DNA strands (Fig. 3a), 24 h after addition of the upper four trigger DNA strands, still a substantial fraction of 30% of fluctuating structures was observed. This shows that structures can return from a fully transformed to a fluctuating state (Supplementary Fig. 13).

Interestingly, for Positions 2-4, the reversible transformations seemed to occur quasi-simultaneously at the different anti-junctions (Fig. 3a, and Supplementary Fig. 11). To quantify the extent of this correlated behavior, we introduced the coupling parameter *C* (Supplementary Fig. 14) that reports on the time two anti-junctions spend in the same conformation (untransformed or transformed) compared to the time they spend in differing conformations. A maximum value of *C* = 1 corresponds to a DNA origami array with maximally coupled junctions in which only fully correlated fluctuations occur. The closer the value is to *C* = 0, the larger is the time the studied junctions spend in differing conformations and the smaller is the extent of coupling. Systems with *C* > 0.95 are considered fully coupled.

Figure 3d-g show the coupling distributions for the transformation of DNA origami arrays with FRET probes at the strongly coupled Positions 2 and 4, and at the less coupled Positions 1 and 3 upon addition of all five and only the upper four trigger DNA strands. In the coupled system, 67% and 56% of all structures exhibited perfect coupling for the transformation upon addition of five and four trigger DNA strands, respectively, indicating that in many structures also all fluctuations are fully correlated. The coupling distribution of the less coupled systems also feature a small distinct peak at *C* > 0.95 (highlighted by the orange bar, 8% and 23% for the addition of five and four trigger DNA strands, respectively) and an additional larger left-skewed distribution close to *C* = 0. Thus, the majority of all systems exhibit largely uncoupled behavior. The coupling distributions of all other studied systems are shown in Supplementary Fig. 15 and are in good agreement with the proposed transformation reaction cascade starting at Position 1 and progressing to Positions 2-4 at which strongly coupled behavior was observed.

By reducing the number of added trigger DNA strands from five to the upper four, the untransformed conformation was destabilized less, resulting in a slower transformation reaction (Fig. 3c). The destabilization of the transformed conformations yielded reversible transformations at 37 °C (Figs. 3h, i) as described by the energy landscape in Fig. 3j.

To further study the extent of the coupling between the transformation steps, we additionally recorded the fluctuations occurring upon the addition of only the upper four trigger DNA strands at Position 2 and Position 4 with a higher temporal resolution of 200 ms. Even with this fivefold improvement in resolution, the transformations at the studied positions still occurred simultaneously, reinforcing the assumption of a strongly coupled system across distal sites (Supplementary Fig. 16).

### Decoupling of anti-junctions in DNA origami array structures by introduction of artificial activation energy barriers

**Fig. 4:**
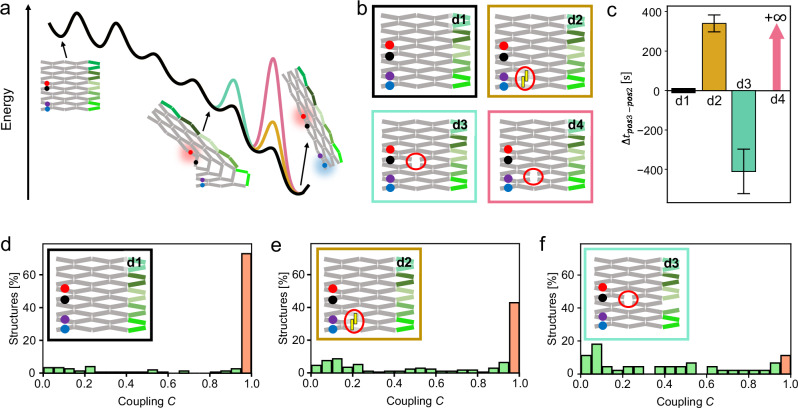
Temporal decoupling of different steps in the transformation reaction by artificially introducing energy barriers. **a**, **b** Mechanisms used to engineer the energy landscape. d1 corresponds to the unmodified reference, d2 to a system with a locking unit incorporated and d3 and d4 to systems with missing central anti-junctions. **c** Lag times for the transformation to progress from Position 2 to Position 3 upon addition of all five trigger DNA strands in the systems shown in (**b**). Error bars represent the standard deviation of the Gaussian fit of the corresponding time difference histograms. For designs in which the majority of structures exhibited a time delay between the transformation at the different positions, only non-perfectly coupled structures with $\triangle t\ne 0\,{{{\rm{s}}}}$ were considered. All plots show the mean values and standard errors of Gaussian fits to the corresponding time difference distributions. **d**–**f** Corresponding coupling histograms. The fraction of structures exhibiting full coupling (*C* > 0.95) is indicated by an orange bar.

With a deeper understanding on the transformation mechanism, we then aimed for modulating the transformation reaction via selective decoupling of anti-junctions. First, we weakened the coupling between individual positions, i.e., between Positions 2 and 3, by engineering the energy landscape at the corresponding step to introduce a heightened activation energy barrier (Fig. 4a). In addition to an unmodified reference (Design 1), three DNA origami arrays bearing FRET probes at Positions 2 and 3 were assembled (Fig. 4b, Supplementary Data 1). In the second array, a locking mechanism was introduced. The mechanism consists of two complementary DNA strands protruding from the origami surface which are in close proximity in the untransformed and further apart in the transformed conformation. Hybridization of the two strands thus stabilizes the untransformed conformation and increases the energy necessary to induce the transformation at the corresponding position (Design 2). For the third and fourth array (Designs 3 and 4), staple strands around positions of central anti-junctions in the structure were left out during assembly of the structures (Supplementary Data 1).

The time lags between the transformation occurring at Positions 2 and 3 upon addition of all five trigger DNA strands are shown in Fig. 4c for all four designs (Supplementary Fig. 17 for time difference histograms). The corresponding coupling distributions are shown in Figs. 4d–f. In Design 1, the transformation at Positions 2 and 3 was strongly coupled (Fig. 4d) with *C* > 0.95 for 73% of all structures. In Design 2, the extent of coupling was reduced and only 40% of all structures exhibited a coupling of *C* > 0.95 (Fig. 4e). This tendency became even more pronounced for Design 3, for which only 12% of structures exhibited a coupling of *C* > 0.95. In the uncoupled structures of Design 2, the transformation preferentially occurred first at Position 2 before progressing to Position 3. In Design 3, the order of events was reversed, and the transformation occurred at Position 3 before progressing to Position 2. For Design 4, the transformation stopped after Position 2 such that it did not progress to Position 3 at all, rendering the global transformation incomplete.

The data shows that the energy landscape of the transformation reaction could be specifically tailored to selectively weaken the coupling between Position 2 and 3 to different extents in Designs 2-4. We concluded that the incorporation of locking units (Design 2) and leaving out staple strands (Design 3, 4) at positions transforming at different steps in the transformation reaction cascade form efficient tools to engineer different extents of coupling and time delays within cascade systems.

Using Design 2, we additionally studied the energy transport efficiency of the transformation reaction at the position of the locking unit. By stepwise increasing the number of hybridizing base pairs of the locking unit (Supplementary Data 1), we systematically varied the hybridization energy of the unit. Quantification of the number of structures transforming at Position 3 within 25 min revealed a 50% transport efficiency for hybridization energy of 16.0 ± 0.5 kcal/ mol (not considering possible effects of binding the locking unit to the DNA origami scaffold, Supplementary Fig. 18).

Besides quantifying how much energy is transferable at a specific step in the transformation reaction, we also demonstrated the transfer of a cargo DNA strand to the environment. Based on the principle of allosteric inhibition, we designed a cargo release unit to which a cargo DNA strand binds in the untransformed conformation. Upon binding of the trigger DNA strands to the transformation origami and the subsequent transformation of the cargo release unit, the binding of the cargo DNA strand to the unit is weakened, resulting in its release. In this process, the mechanical energy of the conformational change is reconverted to chemical energy to dehybridize the cargo DNA strand from the structure–tens of nanometers away from the initial activation site (Supplementary Figs. 19, 20, Supplementary Note 1).

## Discussion

In conclusion, we established a double-FRET single molecule assay to reveal insights into the allosteric transformation reaction cascade of reconfigurable DNA origami arrays. The assay allowed zooming in into single steps of the cascade process, making it possible to characterize the transformation reaction cascade, including intermediates. For our DNA origami array model structure, the energy landscape predominantly depends on the number of open anti-junctions of the intermediates. As such, the first few steps are accompanied by comparably high activation energies, whereas all the following steps occur quasi-simultaneously. Our assay allowed us to define strategies to tailor the transformation reaction cascade both globally and at pre-defined steps. The incorporation of different locking elements into the structure introduces artificial energy barriers, resulting in weakened coupling between selected intermediates, which, in the extreme, leads to altered transformation pathways or incomplete transformations.

The principal findings should be applicable to more complex DNA origami array systems. Such systems could feature different proximity-induced operations. Exemplarily, we demonstrated a cargo DNA strand released at a predefined step in the transformation reaction cascade. Combining the transformation reaction cascade with its intrinsic allosteric control, the addressability of the DNA origami approach, and the findings revealed by our double-FRET single molecule assay highlights the potential of DNA origami arrays as a universal platform to engineer spatially controlled reactions for information and energy transfer. In addition to the prototypical allosteric transfer of spatial information, we added the dimension of temporal control as timing between certain elements could be engineered. Overall, we envision that further developing these approaches will pave the way for DNA origami array systems being used as a platform for programmable, artificial reaction networks containing elements such as cooperativity and anti-cooperativity^28^, rows of logical gating^29,30^ as well as signal amplification^31^ and transduction over several tens of nanometers.

## Methods

### Synthesis of DNA origami arrays

DNA origami structures were designed using the open-source software caDNAno2^32^ and assembled and purified using published protocols^33^. For the exact sequences of all unmodified and modified DNA staple strands used to fold the DNA origami structures see Supplementary Data 1. DNA staple strands were purchased from Eurofins Genomics GmbH (Germany) and Integrated DNA Technologies (USA).

For DNA origami folding, 25.0 μL of in house produced p1800 scaffold at 100 nM were mixed with 3.4 μL of unmodified staples and 8.6 μL of modified staples pooled from 100 μM original concentration. Briefly, 5.0 μL of 10 × TAE buffer (400 mM Tris, 400 mM acetic acid, 10 mM EDTA, pH 8), 6.0 μL of 100 mM MgCl_2_ and 7.0 μL water were added and the mixture was heated to 65 °C in a thermocycler. The solution was kept at this temperature for 15 min before being cooled down to 25 °C with a temperature gradient of – 1 °C min^−1^. Folded DNA origamis were purified from excessive staple strands by gel electrophoresis. All gels were ran using a 1.5% agarose gel, 1 × TAE (40 mM Tris, 40 mM acetic acid, 1 mM EDTA, pH 8) containing 12.5 mM MgCl_2_ for 2 hours at 6 V/cm. The target band containing DNA origami was cut from the gel and DNA origami solution extracted from the band via squeezing.

### Atomic force microscopy (AFM) measurements

The AFM imaging was carried out on the Multimode VIII system (Bruker). 2 µL of the sample was deposited onto freshly cleaved mica surface. The sample area was filled with 80 µL 1 × TE buffer with 10 mM MgCl_2_. The sample was imaged in liquid mode using commercial tips (SNL-10, Bruker). The imaging results were analyzed with Nanoscope analysis (Bruker).

### Sample preparation on the coverslip for single-molecule widefield measurements

Adhesive SecureSeal^TM^ Hybridization Chambers (2.6 mm depth, Grace Bio-Labs, USA) were glued on microscope coverslips (24 mm × 60 mm, 170 μm thickness, Carl Roth GmbH, Germany). 1 M KOH was added to the chambers, incubated for 1 h and washed with 1 × PBS buffer three times. The chambers then were incubated with BSA-Biotin (0.5 mg/mL in 1 × PBS, Sigma Aldrich, USA) for 10 min to passivate the surface and washed with 150 μL 1 × PBS buffer three times. The surfaces were then incubated with NeutrAvidin (0.25 mg/mL in 1 × PBS, Thermo Fisher, USA) for 10 min and then washed three times with 150 μL 1 × PBS buffer. DNA origami structures were then immobilized onto the surfaces of the chambers via biotin-neutrAvidin interactions using a biotinylated DNA staple incorporated in the unused scaffold loop of the structures during folding. For this, 150 μL of the DNA origami sample solution diluted to ~10 pM in 1 × TE buffer containing 750 mM NaCl was incubated in the chambers for 5 min and the chambers then washed with 150 μL 1 × TE buffer containing 750 mM NaCl for three times to remove residual unbound DNA origami. In order to minimize photo bleaching and photoblinking, a reducing and oxidizing buffer system (1 × TAE, 2 mM Trolox/Troloxquinone, 12.5 mM MgCl_2_)^34^ in combination with an oxygen scavenging system (12 mM protocatechuic acid (PCA), 56 μM protocatechuate 3,4-dioxygenase (PCD), 1% glycerol, 2 mM Tris-HCl, 1 mM KCl, 20 μM Na_2_EDTA·2H_2_O) was added prior the measurement.

### Loading of the cargo release unit

The cargo release unit was loaded with an ATTO542 labeled cargo DNA strand by incubating surface immobilized origami structures with 100 nM cargo strand in 1 × TAE containing 12.5 mM MgCl_2_ for 10 min. To remove excess cargo DNA strands, samples were washed three times with 150 μL 1 × TE buffer containing 750 mM NaCl and then prepared for imaging.

### DNA origami transformation procedure

For the transformation of DNA origami structures, an excess of trigger DNA strands (50 nM) were added to photostabilized DNA origami sample chambers at 37 °C. Immediately after addition of the trigger strands, the sample chambers were sealed and the DNA origami imaged.

### Wide-field measurements

The data acquisition of single molecule trajectories was realized with the commercial Nanoimager from Oxford Nanoimaging Ltd. At 532 nm, a 1000 mW laser was used to excite the ATTO542 dye, with a relative power-level set to 9%. At 638 nm, a 1100 mW was used to excite the ATTO647N dye with a relative power-level set to 18%. In order to improve the signal-to-background ratio, the wide field illumination was set to TIRF-illumination. In the emission, spectral filtering is applied to separate the fluorescence from scattered excitation light (685/40 filter for the red detection channel and 585/70 filter for the green detection channel). Data acquisition was initialized by activating the lasers and taking a frame of 100 ms every second separately for both excitation lasers (with a time lag of 0.5 s between them) over a measurement period of 25 min. Measurements were carried out at 37 °C.

### Data analysis

Data processing and analysis of time-lapse movies was realized using custom-written Python scripts. Briefly, the acquired movies were first drift corrected using DNA origami structures carrying fluorophores which were in their fluorescent state throughout the whole measurement as fiducial markers. Spots appearing during the measurement were detected from the drift-corrected movies, and dual-color background-subtracted fluorescence intensity transients of those spots were extracted. To determine transformation times and coupling of single structures, the corresponding transients were fitted using a Hidden Markov model (HMM). Two levels corresponding to the untransformed and transformed state of the structure were defined. Transformation times were defined as the time a structure switches from its untransformed state to its transformed state and subsequently remains in its transformed state for at least 10 s for the first time. They were extracted from the fitted HMM transients. For the calculation of the Coupling between different positions in a structure, transformations state occupancy density plots were created from the dual-color HMM transients. As the ATTO647N and the ATTO542 fluorescence transients were recorded with a time lag of 0.5 sec between them, data points measured in the frame directly before and directly after intensity jumps as determined by the HMM fits were excluded to not artificially weaken the Coupling. The further workflow for calculating the Coupling is given in Supplementary Fig. 11.

For determining the fraction of fluorophores experiencing photobleaching over the 25 min measurement period, all transients were considered. For all further transient analyses, only transients in which both fluorophores turned into their fluorescent state were considered.

## Supplementary information

Supplementary Information

Peer Review File

Description of Additional Supplementary Files

Supplementary Data 1

## Data Availability

The experimental data generated in this study have been deposited in the zenodo database under accession code 10.5281/zenodo.12155916. Data supporting the findings of this manuscript are also available from the authors upon request.

## Code availability

Custom code used for analysis in this study has been deposited in the zenodo database under accession code 10.5281/zenodo.12155916.
