# Supplementary material for: Controlled mechanochemical coupling of anti-junctions in DNA origami arrays
Source: Nat Commun. 2024 Sep 10;15:7894. doi: 10.1038/s41467-024-51721-y (PMC11387415; doi:10.1038/s41467-024-51721-y)
Supplement: Supplementary file 1 — Supplementary Information [file 41467_2024_51721_MOESM1_ESM.pdf]

Supplementary Information

Controlled mechanochemical coupling of anti-junctions in DNA origami arrays

Fiona Cole [1,2,7], Martina Pfeiffer [1,2,7], Dongfang Wang [3,4,5,6,7], Tim Schröder [1,2], Yonggang Ke [3,4*] and Philip Tinnefeld [1,2*]

[1] Department of Chemistry, Ludwig-Maximilians-Universität München, Butenandtstr. 5-13, 81377 München, Germany

[2] Center for NanoScience, Ludwig-Maximilians-Universität München, Schellingstraße 4, 80799 München, Germany

[3] Wallace H. Coulter Department of Biomedical Engineering, Emory University, Atlanta, GA 30322, USA

[4] Georgia Institute of Technology, Atlanta, GA 30322, USA

[5] School of Biomedical Engineering, University of Science and Technology of China, , Suzhou 215123, China

[6] Suzhou Institute for Advanced Research, University of Science and Technology of China, Suzhou 215123, China

[7] These authors contributed equally

[*] Correspondence: philip.tinnefeld@cup.uni-muenchen.de, yonggang.ke@emory.edu

Supplementary Figures

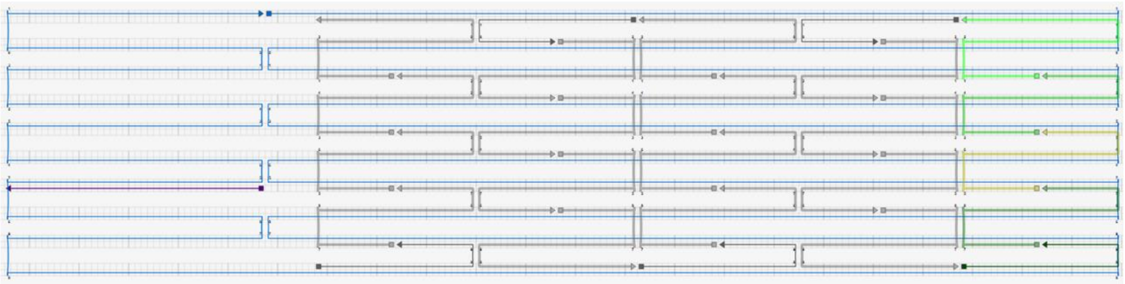

Supplementary Figure 1. Design of the 5 × 2.5 reconfigurable DNA origami array model structure in its untransformed conformation. Blue, gray, purple and green lines represent the scaffold strand, core DNA staple strands of the structure, biotin staple strands and trigger DNA strands, respectively. By labeling one of the staple strands with biotin, we use it as anker point for surface immobilization via biotin-neutravidin interactions. This allows us to avoid directly tethering the core structure (gray staples) to the surface which could possibly affect the transformation process. Trigger DNA strand 1 has a length of 65 base pairs and trigger DNA strands 2-4 all have the same length of 52 base pairs. Trigger DNA strand 5 is shorter, consisting of only 39 base pairs. As the transformation process starts either at the upper right (trigger 1) or lower right corner (trigger 5), this asymmetry induces a preferential transformation starting point. The longer length of trigger 1 compared to trigger 5 results in the transformation preferentially starting from the upper right corner.

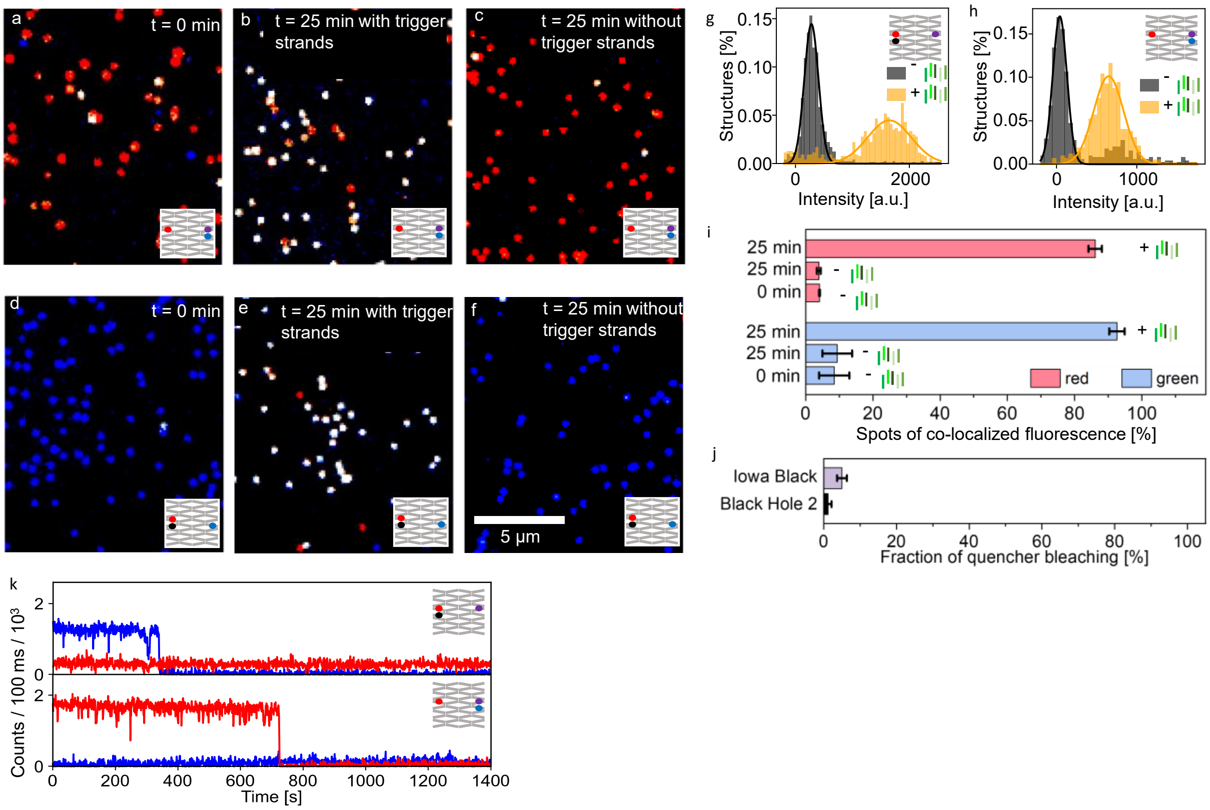

Supplementary Figure 2. Transformation of reconfigurable DNA origami array structures on the single-molecule level. (a-f) Exemplary TIRF images of DNA origami array with (a) the green dye-quencher pair and a red co-localization dye and (b) the red dye-quencher pair and a green co-localization dye incorporated after 0 min incubation, after 25 min incubation (b,e) with all five trigger DNA strands and (c,f) without trigger DNA strands. (g,h) Intensities of the FRET probe in DNA origami arrays prior (grey) and after 25 min (orange) incubation with all five trigger DNA strands for both FRET probes. (i) Fraction of spots of co-localized red and green fluorescence obtained for TIRF images of structures with the red and the green dye-quencher pair as well as a co-localization dye incorporated after 0 min and 25 min of incubation with and without of all five trigger DNA strands. The fraction of spots of co-localized red and green fluorescence indicates the transformation yield as determined with the FRET probes. (j) Fraction of quencher molecules photobleached during the measurement period of 25 min. The fractions were extracted from the intensity transients recorded from the images shown in (a) and (d). (k) Exemplary intensity transients recorded from the images shown in (a) and (d) indicating the absence of quencher blinking. Error bars in (i,j) represent the standard error of at least 200 structures.

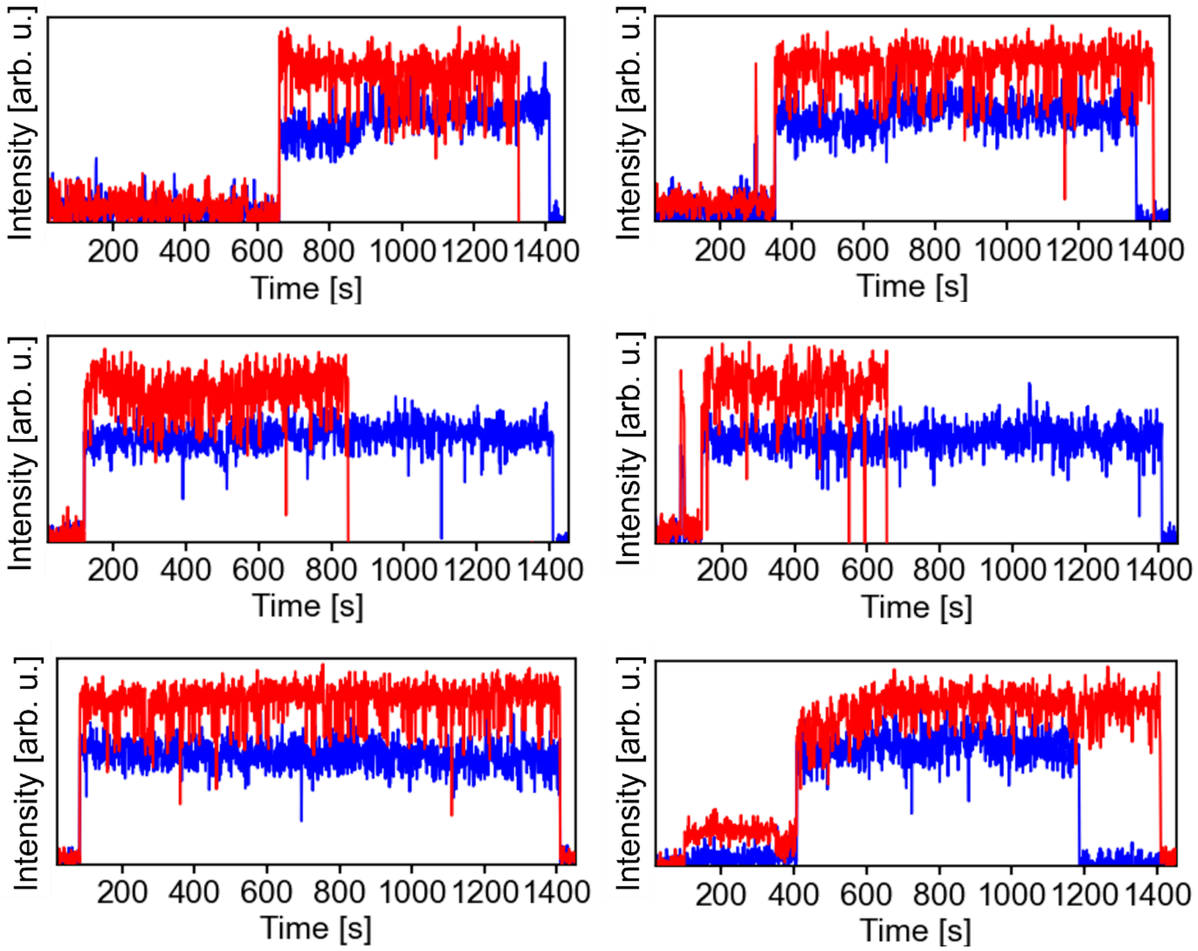

Supplementary Figure 3. Representative single-molecule fluorescence intensity transients of DNA origami array structures with the red and the green FRET probe at Position 4 and Position 2, respectively, recorded after addition of all five trigger DNA strands at 0 s. Fluorescence of ATTO647N is shown in red, fluorescence of ATTO542 in blue. In addition to a sudden jump in fluorescence intensity corresponding to the transformation of the studied position also short dips in fluorescence intensity are observed in the raw data exemplified in Figure S3-S11. While uncorrelated fluctuations can be easily explained by photophysics of the used fluorophores and quenchers, some aspects such as correlated intensity jumps of both dyes simultaneously (e.g. Figure S5 upper left) are sometimes more difficult to interpret. Such effects however occur too infrequently to be included in the statistical analysis.

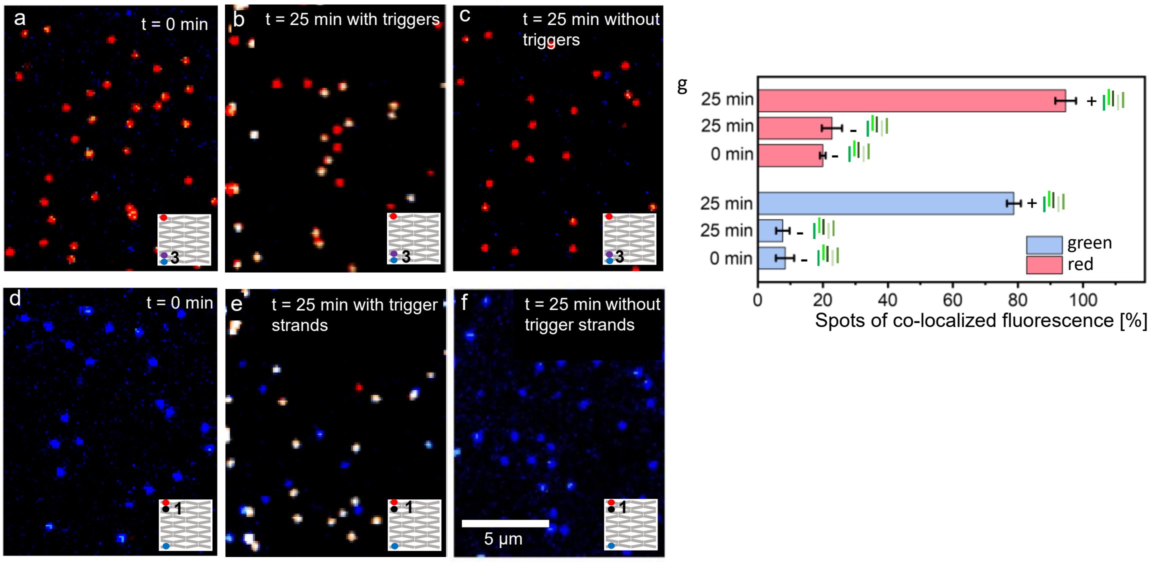

Supplementary Figure 4. Additional FRET probes at Position 1 and Position 3. (a-f) Exemplary TIRF images of DNA origami array with (a) the green dye-quencher pair at Position 3 and a red co-localization dye and (b) the red dye-quencher pair at Position 1and a green co-localization dye incorporated after 0 min incubation, after 25 min incubation (b,e) with all five trigger DNA strands and (c,f) without trigger DNA strands. (g) Fraction of spots of co-localized red and green fluorescence obtained for TIRF images of structures with the red and the green dye-quencher pair as well as a co-localization dye incorporated after 0 min and 25 min of incubation with and without of all five trigger DNA strands. The fraction of spots of co-localized red and green fluorescence indicates the transformation yield as determined with the FRET probes. Error bars in (g) represent the standard error of at least 200 structures.

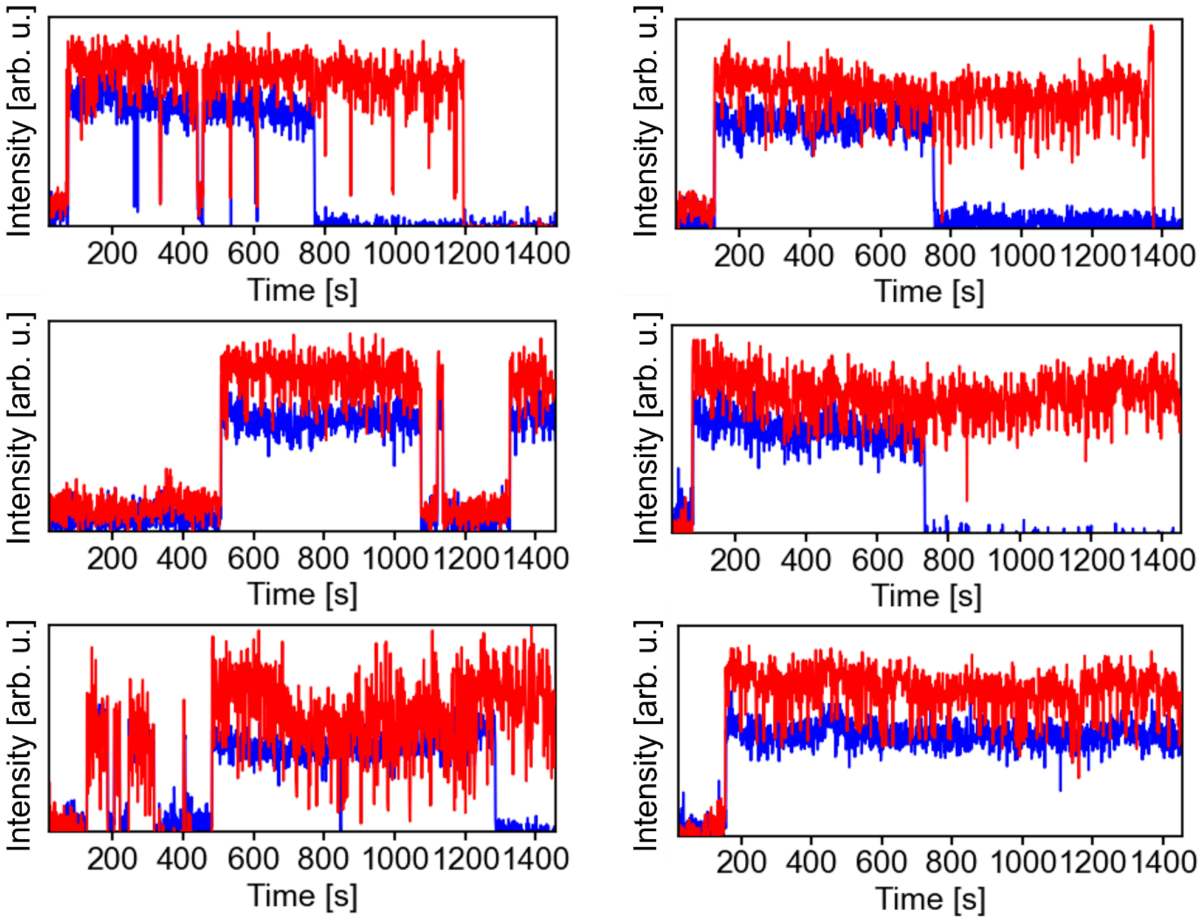

Supplementary Figure 5. Representative single-molecule fluorescence intensity transients of DNA origami array structures with the red and the green FRET probe at Position 2 and Position 3, respectively, recorded after addition of all five trigger DNA strands at 0 s. Fluorescence of ATTO647N is shown in red, fluorescence of ATTO542 in blue.

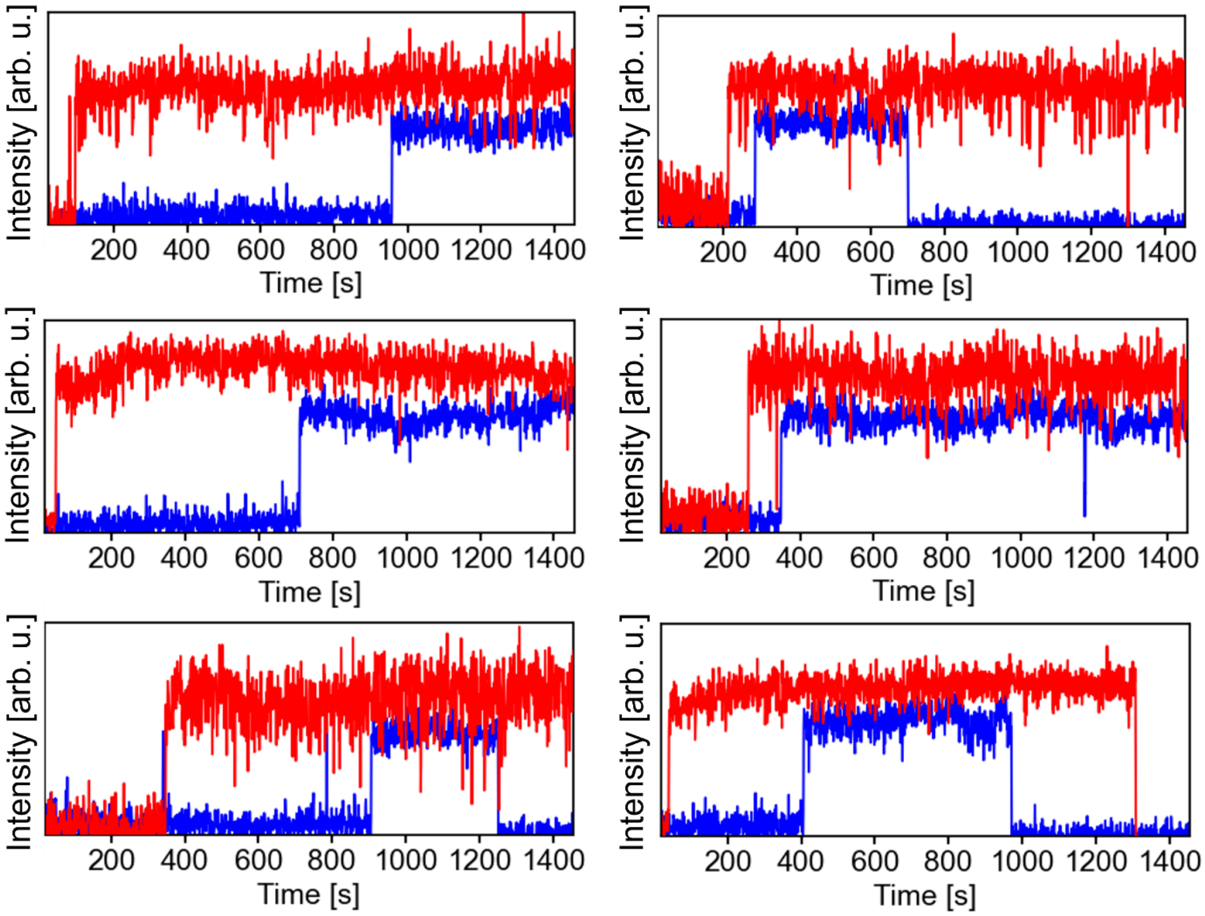

Supplementary Figure 6. Representative single-molecule fluorescence intensity transients of DNA origami array structures with the red and the green FRET probe at Position 1 and Position 3, respectively, recorded after addition of all five trigger DNA strands at 0 s. Fluorescence of ATTO647N is shown in red, fluorescence of ATTO542 in blue.

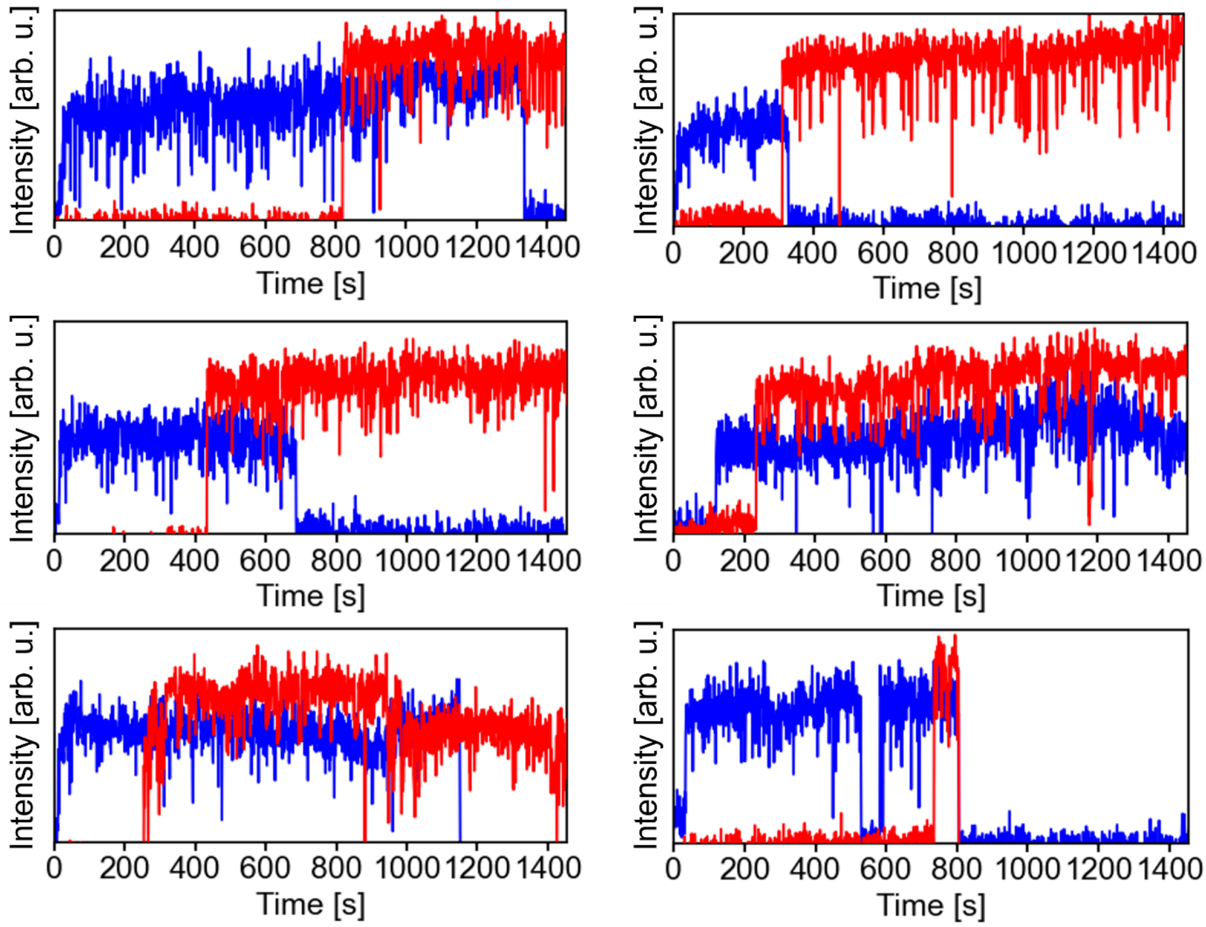

Supplementary Figure 7. Representative single-molecule fluorescence intensity transients of DNA origami array structures with the red and the green FRET probe at Position 2 and Position 1, respectively, recorded after addition of all five trigger DNA strands at 0 s. Fluorescence of ATTO647N is shown in red, fluorescence of ATTO542 in blue.

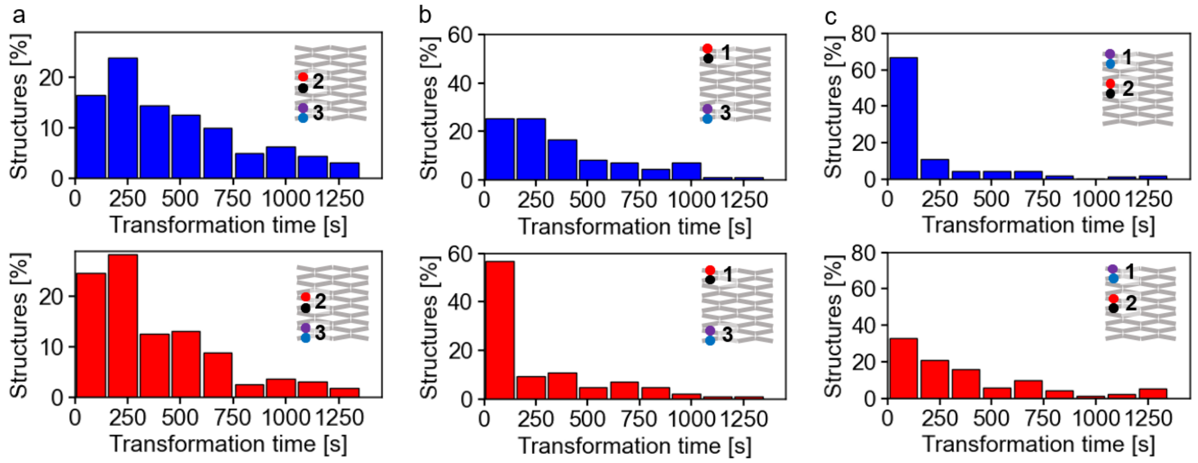

Supplementary Figure 8. Transformation times at the position of the green dye-quencher pair (upper row) and at the position of the red dye-quencher pair (lower row) after addition of all five trigger DNA strands for DNA origami array structures with the dye-quencher incorporated at different positions. (a) Position 2 and 3, (b) Position 1 and 3 and (c) Position 1 and 2.

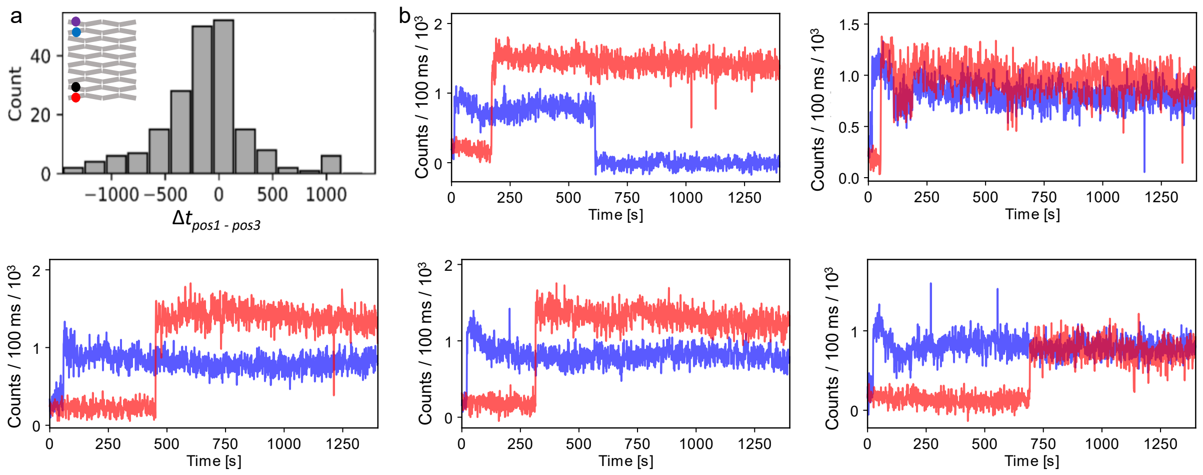

Supplementary Figure 9. Transformation process of DNA origami array with the green and red FRET probes at switched positions in comparison to Figure 2f. The green dye-quencher pair was placed at Position 1 and the red dye-quencher pair at Position 3. (a) Time difference between the transformation occurring at the positions of the green and red dye-quencher pairs positioned at Position 1 and Position 3, respectively. As for the design with the FRET probes placed at the switched positions in Figure 2f, the transformation preferentially first occurs at Position 1, confirming that this tendency was caused by the structure itself and not by differing interactions of the red and green FRET probes with DNA (e.g. stickiness).[1] (b) Representative single-molecule fluorescence intensity transients of a surface-immobilized DNA origami array structures recorded after addition of all five trigger DNA strands at 0 s. Fluorescence of ATTO647N is shown in red, fluorescence of ATTO542 in blue.

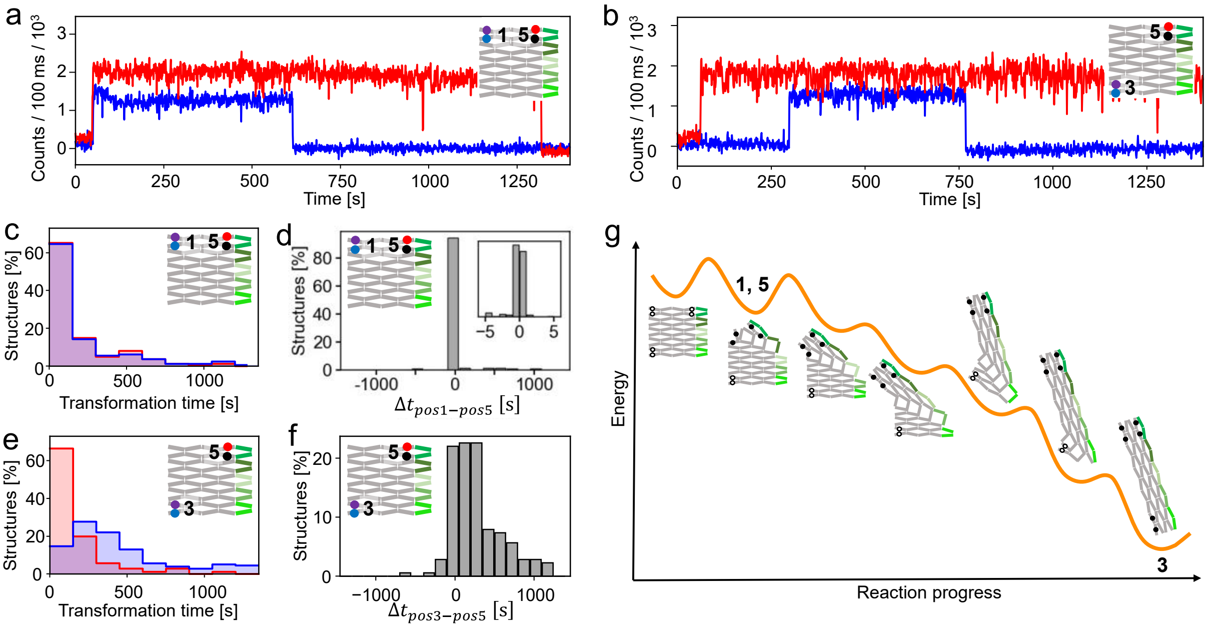

Supplementary Figure 10. Transformation at Position 5 after addition of all five trigger DNA strands at 0s. (a-b) Representative single-molecule fluorescence intensity transients of DNA origami array structures with the red and green FRET probe at (a) the newly introduced Position 5 and Position 1 and (b) Position 5 and Position 3, respectively. (c-f) Analysis of the transformation times. (c,e) Transformation times after addition of five trigger DNA strands at the positions of the red and the green FRET probe. (d,f) Time differences between the transformation occurring at the positions of the green and red FRET probes. The transformation occurs simultaneously at Position 1 and Position 5 and time-delayed at Position 3. (g) Proposed, simplified sketch of the energy landscape of the transformation reaction which is in agreement with the measured time difference distributions. The intermediates at which the studied positions first transform are marked with numbers.

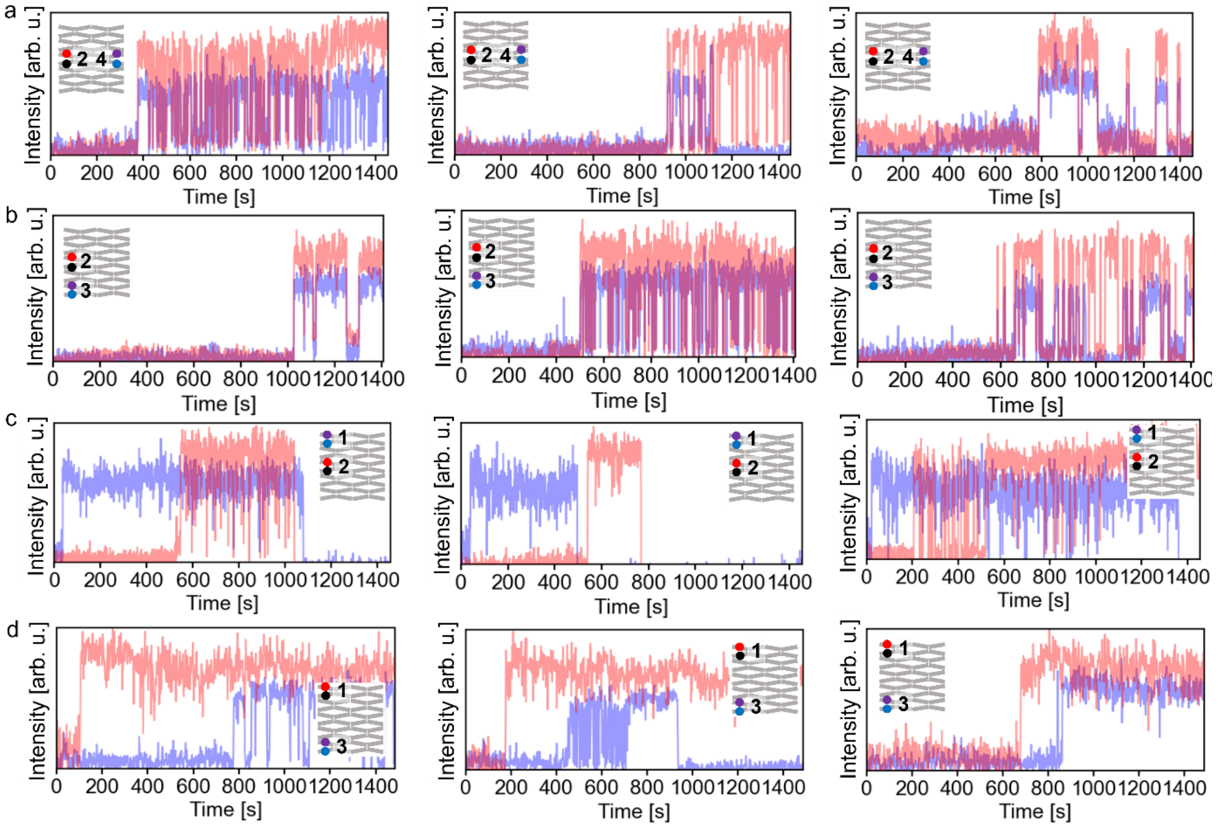

Supplementary Figure 11. Representative single-molecule fluorescence intensity transients of surface-immobilized DNA origami arrays with the red and the green dye-quencher pair incorporated at different positions. (a) Position 4 and Position 2, (b) Position 3 and Position 2, (c) Position 1 and Position 2, (d) Position 1 and Position 3 recorded after addition of the upper four trigger DNA strands at 0 s. Fluorescence of ATTO647N is shown in red, fluorescence of ATTO542 in blue.

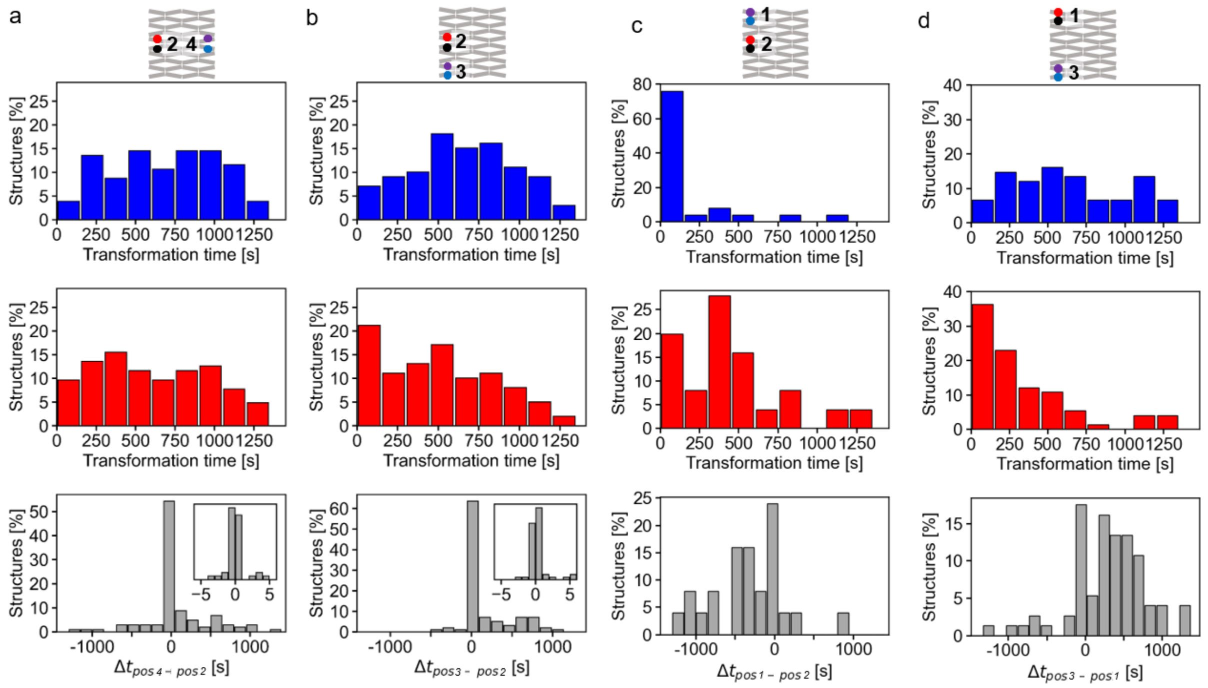

Supplementary Figure 12. Transformation times at the position of the green dye-quencher pair (upper row) and at the position of the red dye-quencher pair (middle row) and time differences between the transformation at the position of the green dye-quencher pair and the position of the red-dye quencher pair after addition of the upper four trigger DNA strands with the dye-quencher incorporated at different positions. (a) Position 4 and 2, (b) Position 3 and 2, (c) Position 1 and 2, (d) Position 3 and 1.

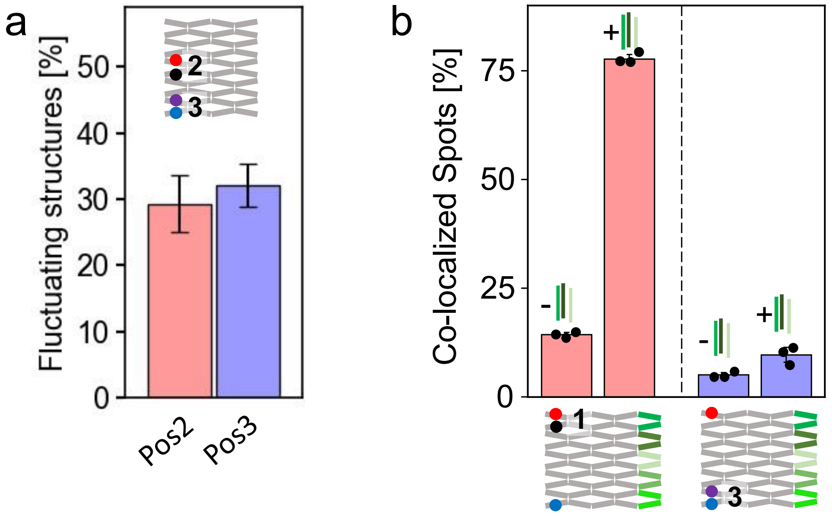

Supplementary Figure 13. Continuous fluctuations and incomplete transformation when reducing the number of trigger DNA strands. (a) Fraction of structures showing intensity fluctuations after 24 h incubation with four trigger DNA strands. Error bars represent the standard error of 82 structures. (b) Fraction of co-localized spots of red and green fluorescence in TIRF images of DNA origami arrays (left) with the red dye-quencher pair incorporated at Position 1 and a green co-localization dye incorporated at Position 3 and (right) with the green dye-quencher pair incorporated at Position 3 and a red co-localization dye incorporated at Position 1 prior and after incubation with the upper three trigger DNA strands for 25 min. For the second origami array, only a small increase in the number of co-localized structures upon incubation with the trigger DNA strands was observed. This indicates incomplete transformation not progressing through Position 3 upon addition of only the upper three trigger DNA strands. Error bars represent the standard deviation in the fractions calculated from three TIFR images (at least 250 single nanostructures per image).

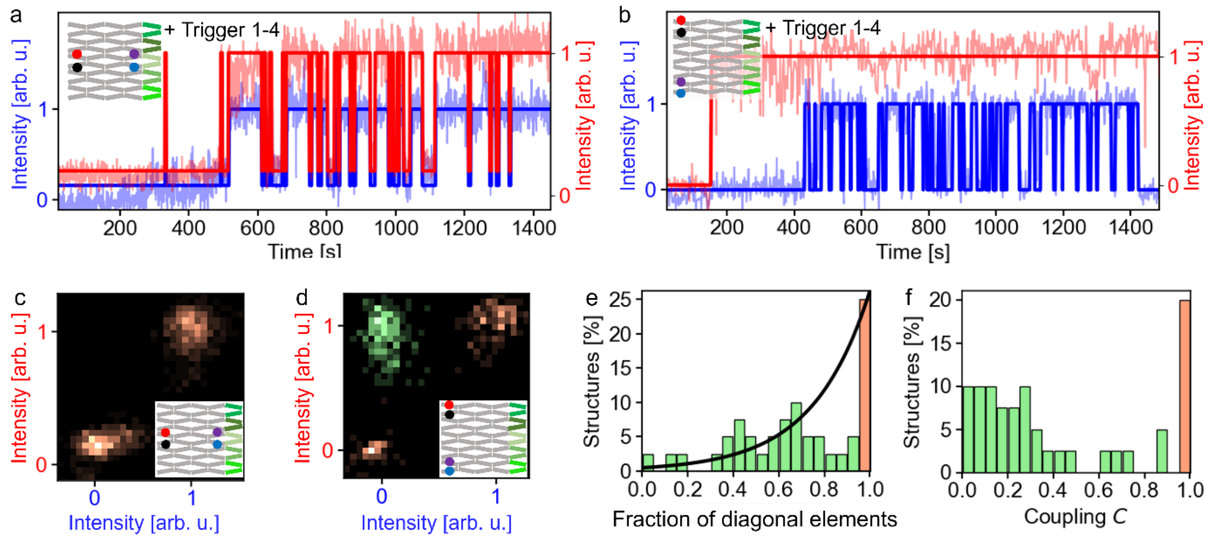

Supplementary Figure 14. Determination of the Coupling C. (a, b) Representative single-molecule fluorescence intensity transients of DNA origami arrays with the red and the green FRET probe incorporated at (a) Position 2 and 4 and (b) Position 1 and 3 of ATTO542 fluorescence (blue transients) and ATTO647N fluorescence (red transient) after addition of the upper four trigger DNA strands. The Coupling of the transformation between two positions in a DNA origami structure is calculated using the transformation state occupancy density plots of the corresponding positions in the structure. (c,d) transformation state occupancy density plots for a system exhibiting (c) $C = 1$ and (d) with $C < 0.95$. In these density plots, the fraction of diagonal elements with respect to the total number of both diagonal and off-diagonal elements is determined. (e) Exemplary distribution of the fractions of diagonal elements calculated for the structures shown in (b). The Coupling between two positions in a system is calculated by weighing the fraction of diagonal elements with the exponential distribution $C = \exp\left(-4\,(1 - \mathrm{fraction})\right)$ shown by the black line. This separates perfectly coupled systems ($C = 1$) from slightly uncoupled systems. (f) Resulting Coupling distribution for the data shown in (e). The fraction of structures exhibiting full coupling is indicated by an orange bar.

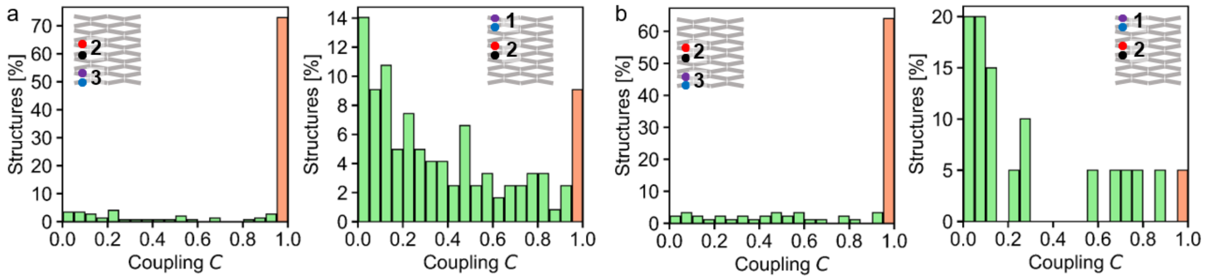

Supplementary Figure 15. Coupling histograms for the dye quencher pairs at different positions upon the addition of different numbers of trigger DNA strands. (a) all five trigger DNA strands and (b) only the upper four trigger DNA strands.

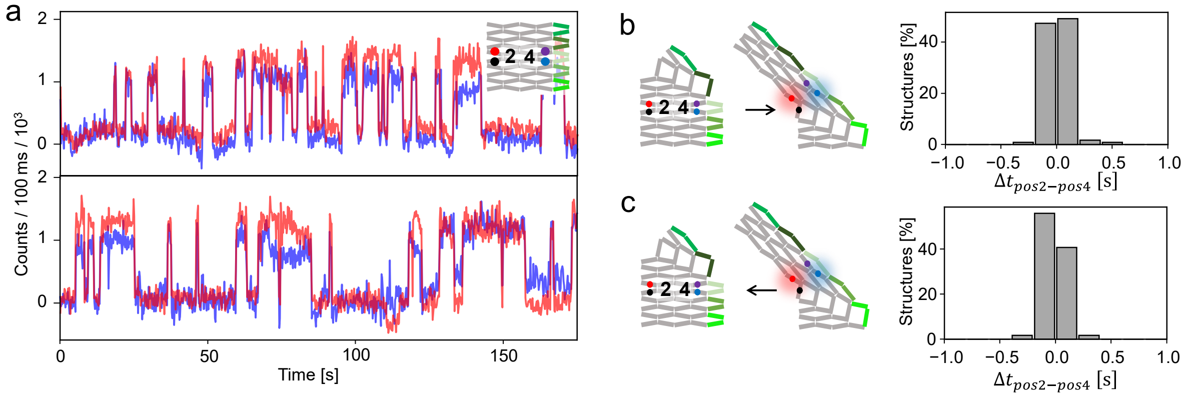

Supplementary Figure 16. Transformation time differences between Position 2 and Position 4 measured with an improved temporal resolution of 200 ms. (a) Representative single-molecule fluorescence intensity transients of DNA origami array structures with the red and green FRET probe at Position 2 and Position 4, respectively. The transients were recorded 10 min after addition of the upper four trigger DNA strands with alternating laser excitation (ALEX) of the green and the red laser (100 ms per frame), resulting in an improved temporal resolution of 200 ms. (b, c) Sketch of (a) the forward and (b) the reverse reaction of the transformation process occurring at both Position 2 and Position 4 (left) and the corresponding time differences between the reactions occurring at the corresponding positions (right). The time difference distributions are binned according to the experimental temporal resolution. They show that both the forward and the reverse transformation occurs in consecutive frames of the ALEX illumination scheme at the studied positions in > 96% of all cases, indicating that the studied transformation steps occur (quasi)simultaneous also at an improved temporal resolution of 200 ms. For calculation of the time difference histograms more than 100 forward and more than 100 reverse fluctuations were analyzed.

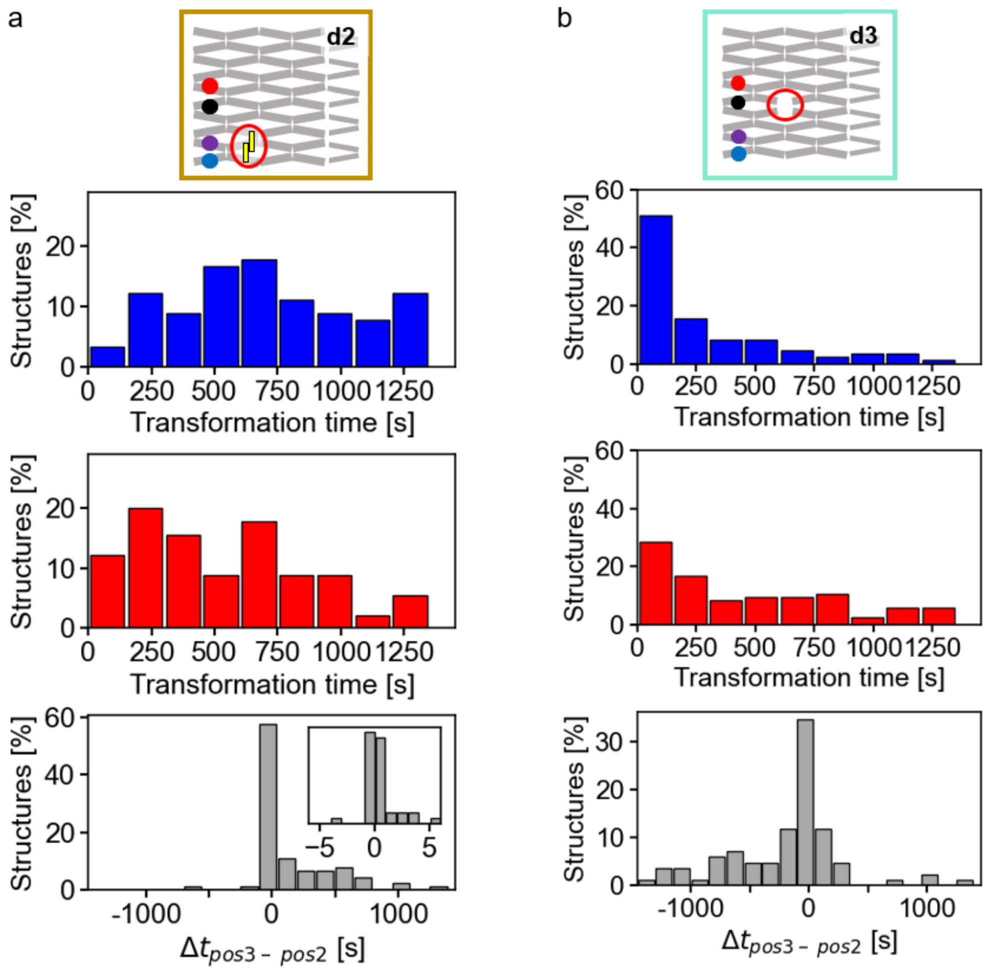

Supplementary Figure 17. Transformation times at the position of the green dye-quencher pair (upper row) and at the position of the red dye-quencher pair (middle row) and time differences between the transformation at the position of the green dye-quencher pair and the position of the red-dye quencher pair after addition of all five trigger DNA strands with the dye-quencher pairs incorporated Position 3 and Position 2 to different DNA origami array constructs. (a) DNA origami arrays with a locking unit incorporated and (b) DNA origami arrays with missing staples at an anti-junction (red circle). For determination of the mean time differences shown in Figure 4c only non-perfectly coupled structures with $\Delta t \neq 0\,s$ were considered.

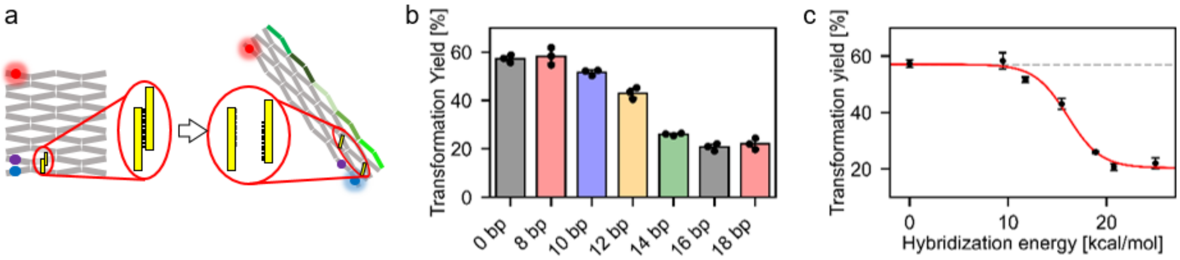

Supplementary Figure 18. Energy transport efficiency at the position of the locking unit. (a) Origami design for determining the energy transport efficiency. We systematically varied the number of hybridizing base pairs of the locking unit and thus its hybridization energy and then determined the transformation yield at the lower left position (Position 3). For colocalization, we additionally placed an AT647N dye at Position 1. (b) Transformation yields after 25 min incubation with five trigger DNA strands at the lower left position (Position 3) for different numbers of hybridizing base pairs in the locking unit. Transformation yields were determined from TIRF images by dividing the number of colocalized red-green spots by the total number of red spots. (c) Transformation yield as a function of the hybridization energy of the locking unit. The data points were fitted by a logistic function (red curve) to determine the hybridization energy at which 50% energy transfer occurs within 25 min as 16.0±0.5 kcal/mol. The grey dashed line represents the fraction of co-localized spots in an origami with a 0 bp locking unit folded in its transformed state. Hybridization energies were estimated using NUPACK without considering possible effects of binding the locking unit to the DNA origami array scaffold.[2] Error bars in (b,c) represent the standard deviation in transformation yield of three TIFR images (at least 300 single nanostructures per image).

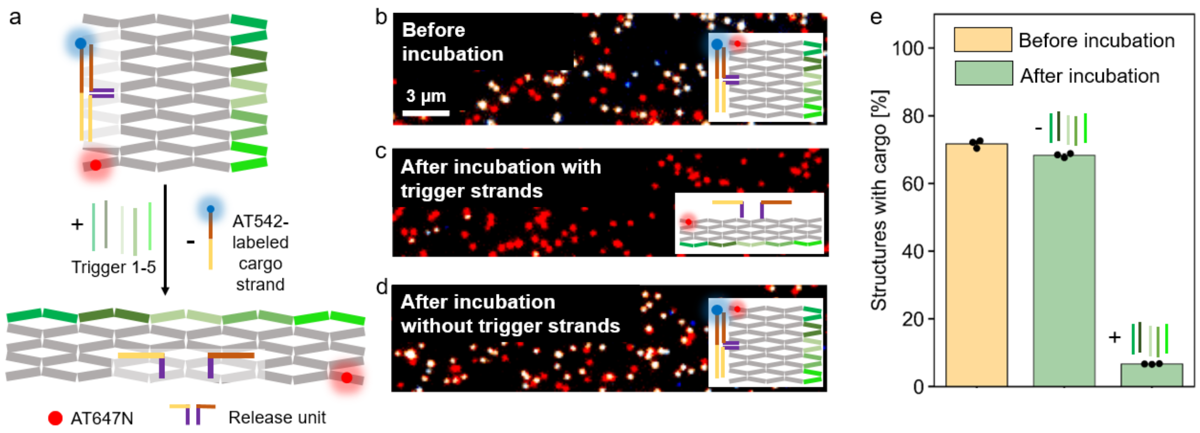

Supplementary Figure 19. Cargo release. (a) Scheme of the procedure of cargo release. (b) TIRF image of the DNA origami array bearing a catching unit after incubation with an ATTO542 labeled cargo strand. (c, d) TIRF images of DNA origami arrays carrying ATTO542 labeled cargo strand after 25 min incubation (c) without and (d) with all five trigger DNA strands. (e) Corresponding fractions of DNA origami array structures carrying a cargo DNA strand. Error bars represent the standard deviation in the fractions calculated from three TIFR images (at least 300 single nanostructures per image).

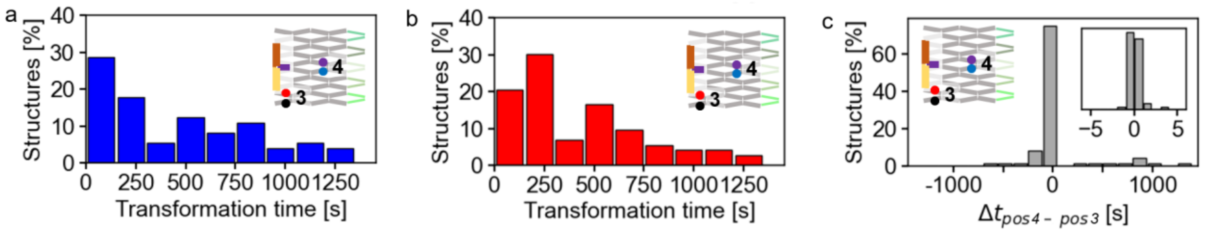

Supplementary Figure 20. Transformation of DNA origami arrays with an incorporated cargo release unit and the green dye-quencher pair incorporated at Position 4 and the red dye-quencher pair at position 3. (a, b) Transformation time at the position of (a) the green dye quencher pair and (b) the red dye-quencher pair. (c) Time difference between the transformation occurring at the positions of the green and red dye-quencher pairs.

Supplementary Notes

Supplementary Note 1. Temporal and spatial controlled proximity-induced reactions in DNA origami array structures. Temporally and spatially controlled proximity-induced reaction cascades could find exciting applications e.g. in a cargo release process. For this, a cargo release unit was positioned on the DNA origami array structure at Position 2.

The cargo release unit is formed by two DNA strands protruding from the DNA origami array on neighboring helices. They contain a 6-nt complementary sequence which forms a stem followed by a 10-nt non-complementary sequence on each strand to which an ATTO542-labelled single-stranded DNA strand containing a 20-nt complementary sequence can permanently hybridize (Supplementary Figure 19a). Due to the opening of the stem, the distance between the arms of the catching unit becomes too large to enable a permanent binding of the cargo strand to both arms and the cargo is released. Thus, the release of the cargo strand could be initiated by addition of trigger DNA strands and performed at the pre-programmed position in the transformation cascade. The unbinding of the cargo strand was detected in single-molecule TIRF images (Supplementary Figure 19b-d) where white spots of co-localized fluorescence of ATTO647N and ATTO542 are attributed to DNA origami structures with a bound cargo release strand while red spots of ATTO647N fluorescence indicate the presence of DNA origami array structures bearing no cargo strand. Blue spots of ATTO542 fluorescence were attributed to non-specific bound cargo strands or DNA origami array structures with a bound cargo and an unlabeled ATTO647N DNA strand. After 25 min incubation with trigger DNA strands the number of co-localized spots decreased drastically (see Supplementary Figure 19c) while only a slight drop in the number of co-localized spots was observed upon incubation without trigger strands (Supplementary Figure 19d). Quantification (Supplementary Figure 19e) confirmed the specific nature of cargo release strand unbinding initiated by the transformation process. Interestingly, the introduction of the cargo release process did not induce a time delay between the FRET probes framing the cargo release unit (see Supplementary Figure 20).

Sequence of the p1800 scaffold used to fold the DNA origami array structure from 5' to 3' end:

TACGAAGAGTTCCAGCAGGGATTCCAAGAAATGGCCAATGAAGATTGGATCAC
CTTTCGCACTAAGACCTACTTGTTTGAGGAGTGCCTGATGAATTGGCACGACCG
CCTCAGGAAAGTGGAGGAGCATTCTGTGATGACTGTCAAGCTCCAATCTGAGG
TGGGCAAATATAAGATTGTTATCCCTATCTAGAAGTACGTCCGCGGAGAACACC
TGCCACCCGATCACTGGCTGGATCTGTTACGCTTGCTGGGTCTGCCTCGCGGC
ACATCTCTGGAGAAACTGCTGTTCGGTGACCTGCTGAGAGTTGCCGATACCATC
GTGGCCAAGGCTGCTAACCTGAAAGATCTGAACTCACGCGGCCAGGGTGAAGT
GACCATCCGCGAATAACTCAGGGAACTGGATTTGTGGGGCGTGGGTGCTGTGT
TCACACTGATCGGCTATGAGGACTCCCAGAGCCGCACCTAGAAGCTGATCAAG
GATTGGAAGGAGCTCGTCAACCAGGTGGGCGACAATATATGCCTCCTGCAGTC
CTTGAAGGACTCACCATACTATAAAGGCTTTGAAGACAAGGTCAGCATCTGGGC
AAGGAAACTCGCCGAACTGGACGATAATTTGCAGAACCTCAACCATATTCGCAG
AAAGTGGGTTTACCTCGAACCATACTTTGGTCGCGGAGCCCTGCCCAAAGAGC
AGACCAGATTCAACAGGGTGGGCGAAGATTTCCGCAGCATCATGACATATATCA
AGAAGGACAATCGCGTCACGCCCTTGACTACCCACGCAGGCATTCTAAACTCA
CTGCTGACCATCCTGGACCAATTGCAGAGATGCCAGCGCAGCCTCAACGAGTT
CCTGGAGGCGAAGCGCAGCGCCTTCCCTCGCTTTAACTTCATCGGAGACGATG
ACCTGCGCGAGATCTTGGGCCAGTCAACCAATTAATCCGTGATTCAGTCTCACC
TCAAGAAGCTGTTTGCTGGTATCAACTCTGGCTGTTTCGATGAGAAGTCTAAGC
ACTATACTGCAATGAAGTCCTTGGAGGGGCAAGTTGTGCCATTCAAGAATAACG
TACCCTTGTCCAATAACGTCGAAACCTGGCTGAACGATCTGGCCCTGGAGATG
AAGAAGACCCTGGAGGCGCTGCTGAAGGAGTGCGTGACAACTAGACGCAGCT
CTCAGGGAGCTGTGGGCCCTTCTCTGTTCCCATCACAGATCTAGTGCTTGGCC
GAACAGATCAAGTTTACCGAAGATGTGGAGAACGCAATTAAAGATCACTCCCTG
CACCAGATTGAGTAACAGCTGGTGAACAAATTGGAGCAGTATACTAACATCGAC
ACATCTTCCGTAGACCCAGGTAACACAGAGTCCGGTATTCTGGAGCTGAAACTG
AAAGCACTGATTCTCGACGGATCCACGCGCCCTGTAGCGGCGCATTAAGCGCG
GCGGGTGTGGTGGTTACGCGCAGCGTGACCGCTACACTTGCCAGCGCCCTAG
CGCCCGCTCCTTTCGCTTTCTTCCCTTCCTTTCTCGCCACGTTCGCCGGCTTTC
CCCGTCAAGCTCTAAATCGGGGGCTCCCTTTAGGGTTCCGATTTAGTGCTTTAC
GGCACCTCGACCCCAAAAAACTTGATTTGGGTGATGGTTCACGTAGTGGGCCA
TCGCCCTGATAGACGGTTTTTCGCCCTTTGACGTTGGAGTCCACGTTCTTTAAT
AGTGGACTCTTGTTCCAAACTGGAACAACACTCAACCCTATCTCGGGCTATTCT
TTTGATTTATAAGGGATTTTGCCGATTTCGGGGTACC

Supplementary References

(1) Jahnke, K.; Grubmüller, H.; Igaev, M.; Göpfrich, K. Choice of fluorophore affects dynamic DNA nanostructures. *Nucleic Acids Res* **2021**, *49* (7), 4186–4195. DOI: 10.1093/nar/gkab201.

(2) Zadeh, J. N.; Steenberg, C. D.; Bois, J. S.; Wolfe, B. R.; Pierce, M. B.; Khan, A. R.; Dirks, R. M.; Pierce, N. A. NUPACK: Analysis and design of nucleic acid systems. *Journal of computational chemistry* **2011**, *32* (1), 170–173. DOI: 10.1002/jcc.21596.
