# Supplementary material for: Controlled mechanochemical coupling of anti-junctions in DNA origami arrays
Source: Nat Commun. 2024 Sep 10;15:7894. doi: 10.1038/s41467-024-51721-y (PMC11387415; doi:10.1038/s41467-024-51721-y)
Supplement: Supplementary file 3 — Description of Additional Supplementary Files [file 41467_2024_51721_MOESM3_ESM.pdf]

Description of Additional Supplementary Files

Title: Supplementary Data 1

Description: Sequences of the DNA oligonucleotides used for DNA origami folding.
