# Supplementary material for: Controlled mechanochemical coupling of anti-junctions in DNA origami arrays
Source: Nat Commun. 2024 Sep 10;15:7894. doi: 10.1038/s41467-024-51721-y (PMC11387415; doi:10.1038/s41467-024-51721-y)
Supplement: Supplementary file 2 — Peer Review File [file 41467_2024_51721_MOESM2_ESM.pdf]

nature portfolio

Peer Review File

Controlled mechanochemical coupling of anti-junctions in DNA origami arrays

Open Access This file is licensed under a Creative Commons Attribution 4.0 International License, which permits use, sharing, adaptation, distribution and reproduction in any medium or format, as long as you give appropriate credit to the original author(s) and the source, provide a link to the Creative Commons license, and indicate if changes were made. In the cases where the authors are anonymous, such as is the case for the reports of anonymous peer reviewers, author attribution should be to 'Anonymous Referee' followed by a clear attribution to the source work. The images or other third party material in this file are included in the article's Creative Commons license, unless indicated otherwise in a credit line to the material. If material is not included in the article's Creative Commons license and your intended use is not permitted by statutory regulation or exceeds the permitted use, you will need to obtain permission directly from the copyright holder. To view a copy of this license, visit http://creativecommons.org/licenses/by/4.0/.

Editorial note: This manuscript has been previously reviewed at another journal that is not operating a transparent peer review scheme. This document only contains reviewer comments and rebuttal letters for versions considered at *Nature Communications*.

REVIEWERS' COMMENTS

Reviewer #1 (Remarks to the Author):

The authors have sufficiently addressed my concerns from the previous round of review. This is really nice work for both novel application of single-molecule fluorescence technique and new insight of the timing between coupled folding steps (which is uniquely available from single-molecule experiments). I recommend publication of the revised manuscript.

Reviewer #2 (Remarks to the Author):

I want to thank the authors for the comprehensive revision of the text, according to the review report. I think this is an excellent work, and I look forward to seeing it soon in its published version.

I have no further requests.

Reviewer #3 (Remarks to the Author):

The revised manuscript has addressed all points I raised during the previous round of review. I support publication.
